# Vertebral pattern variation in the North Sea harbor porpoise (*Phocoena phocoena*) by computed tomography

**DOI:** 10.1002/ar.24524

**Published:** 2020-10-16

**Authors:** Dorien S. Willems, Lonneke L. IJsseldijk, D. Hendrik N. van den Broek, Stefanie Veraa

**Affiliations:** ^1^ Diagnostic Imaging, Department of Clinical Sciences, Faculty of Veterinary Medicine Utrecht University Utrecht The Netherlands; ^2^ Division of Pathology, Department of Biomolecular Health Sciences, Faculty of Veterinary Medicine Utrecht University Utrecht The Netherlands

**Keywords:** anatomy, cetacea, imaging, odontoceti, skeleton, spine

## Abstract

Vertebral series in the harbor porpoise (*Phocoena phocoena*) include cervical, thoracic, lumbar, and caudal. In contrast to studying skeletons from museums, in which small bones can be missed, evaluation of full body computed tomography (CT) scans provides an overview of the vertebral column, while maintaining interrelationship of all structures. The aim of this study was to document variations in vertebral patterning of the harbor porpoise via evaluation of CT images of intact stranded harbor porpoises. The harbor porpoises were divided into age classes, based on developmental stage of reproductive organs on postmortem examination and closure of proximal humeral physis on CT. Numbers of vertebrae per series, fusion state of the syncervical, type of first hemal arch, number of double articulating ribs, and floating ribs were recorded based on CT images. Included in the study were 48 harbor porpoises (27 males and 21 females), which were divided in two age classes (27 immatures and 21 adults). Total vertebral count varied from 63 to 68 with vertebral formula range C7T12‐14L12‐16Cd29‐33. Twenty‐five different vertebral formulas were found, of which C7T13L14Ca30 was the most common (*n* = 8, 17%). Thoracic vertebrae with six, seven, or eight double articulating ribs and zero, one, or two vertebrae with floating ribs were seen. Four different fusion states of the syncervical and four types of hemal arches were recognized. This study showed a great variation in vertebral patterning in the harbor porpoise, with homeotic and meristic variation in the thoracic, lumbar, and caudal vertebral series.

## 1 INTRODUCTION

Classically, vertebrae have been allocated to series (cervical, thoracic, lumbar, sacral, and caudal) by their relative position along the anterior/posterior axis, the morphology of individual vertebrae, and the locations of the limbs (Burke, 2000; Winslow, Takimoto‐Kimura, & Burke, 2007). The family of Phocoenidae, including the harbor porpoise (*Phocoena phocoena)*, is part of the superfamily Delphinoidea of infraorder Cetacea. Cetaceans differ from terrestrial mammals in some aspects of vertebral morphology. Thin plate‐like and fused cervical vertebrae (Galatius & Kinze, 2003) are represented in the so‐called syncervical in some cetaceans, such as the harbor porpoise. In terrestrial mammals, the lumbar series terminates before the sacrum. However, cetaceans have no fused vertebrae that form a sacrum (Cozzi, Huggenberger, & Oelschläger, 2017) and therefore, the sacrum cannot be used to determine the most caudal lumbar vertebra (Narita & Kuratani, 2005). Instead in cetaceans, hemal arches indicate the transition from lumbar to caudal vertebrae (Cozzi et al., 2017).

High‐resolution computed tomography (CT) examinations of complete carcasses provide a detailed image of the bony structures. This facilitates the possibility of volume rendering, thereby creating 3‐dimensional reconstructions of the entire animal or applying multiplanar reconstructions (MPRs) enabling extensive evaluation of the skeleton.

CT imaging also provides detailed information about shape and mineralization of bones. Therefore, age determination in human can be estimated by CT evaluation of the stage of ossification of for example the medial epiphysis of the clavicle (Kreitner, Schweden, Riepert, Nafe, & Thelen, 1998; Zhang, Cheng, Zhao, Dong, & Deng, 2015). Galatius and Kinze (2003) studied the physeal closure pattern of bony structures in skeletons of harbor porpoises and found a highly variable time to full closure of the vertebral epiphyses up to 22 years. Full vertebral physeal closure is considered rare (Galatius & Kinze, 2003). The physeal closure process in the proximal humerus was typically completed in 3‐year‐old harbor porpoises and did not show intersexual difference (Galatius & Kinze, 2003).

Cetaceans have a variable vertebral count (Buchholtz, Wolkovich, & Cleary, 2005; Cozzi et al., 2017; Galatius & Kinze, 2003; Kinze, 2009; Newcomer, Jefferson, & Brownell, 1996), which differs from most Mammalia that have a consistent count of seven cervical and 19–20 thoracolumbar vertebrae (Galis, 1999; Galis et al., 2006; Narita & Kuratani, 2005). In Delphinidae, which is another family in the superfamily Delphinoidae, thoracolumbar count varies in different species between 11 and 16 thoracic vertebrae and between 9 and 33 lumbar vertebrae (Buchholtz et al., 2005; Cozzi et al., 2017; Kinze, 2009; Newcomer et al., 1996). In the family Phocoenidae, vertebral counts for specimens of the *Phocoena spinipinnis* were C7T14L15Ca31–35 (Reyes, 2009) and C7T13L15Ca34 (Buchholtz et al., 2005). Limited documentation of vertebral formula in the harbor porpoise is available, two different vertebral formulas have been documented, including C7T13L14Ca31 (Buchholtz et al., 2005) and C7T12L15Ca30–35 (Galatius & Kinze, 2003), resulting in a thoracolumbar count of 27 in both documentations and showing homeotic variation. Homeotic variation augments count in one section at the expense of an adjacent section, preserving total vertebral count (Buchholtz & Stepien, 2009; Burke, Nelson, Morgan, & Tabin, 1995). When a change in one section changes the total number of vertebrae, this is called meristic variation (Buchholtz & Stepien, 2009).

A disadvantage when studying museum skeletons of harbor porpoises is the possibility of missing small bony structures like the last caudal vertebrae, small rib remnants and bones of hemal arches (Cozzi et al., 2017; Galatius & Kinze, 2003). Application of CT of intact harbor porpoises to evaluate the skeleton resolves this as there is no loss of small bony structures.

The aim of this study was to document variations in vertebral patterning of the harbor porpoise via CT evaluation of intact stranded harbor porpoises.

## 2 MATERIALS AND METHODS

### 2.1 Data collection

Fresh carcasses of stranded harbor porpoises in The Netherlands that were collected for postmortem examination, conducted at the Faculty of Veterinary Medicine of Utrecht University between November 2016 and August 2018, were considered for inclusion in this study, 48 harbor porpoises were eligible, including 27 males and 21 females. Data recorded during postmortem examination for each harbor porpoise included sex, total body length and age class. Total body length (in cm) was measured from the tip of the rostrum to the notch in the fluke, in a straight line next to the body (measurements in Supplementary Data). Immature harbor porpoises were differentiated from adults by assessment of the developmental stage of the reproductive organs during postmortem examination (IJsseldijk, Kik, & Gröne, 2019). In addition, humeral proximal physis closure was assessed on CT images. Bilateral the proximal humeral physes were scored as open, if a partial or complete zone of absent to reduced mineral attenuation was noted uni‐ or bilateral or scored as closed if continuation of the mineral attenuation was present bilaterally. Porpoises were considered immatures if the physis was open and adults if completely closed (CT images in Supplementary Data). One male harbor porpoise was presumed immature based on assessment of the reproductive organs, despite complete proximal physeal mineralization on CT examination. This individual is considered immature for further analysis. All other age class differentiations between adult and immature harbor porpoises were confirmed with humeral physis closure status on CT examination. This resulted in the following division per age class: 27 immatures (16 males, length range 79–128 cm; 11 females, length range 72–134 cm) and 21 adult (11 males, length range 130–150 cm; 10 females, length range 133–167 cm) harbor porpoises.

CT equipment was protected with plastic sheets prior to positioning the harbor porpoises. The porpoises were positioned in ventral recumbency on the table of a 64‐slice sliding gantry CT scanner (Somatom Definition AS, Siemens AG, München, Germany). Raw data of the entire harbor porpoise were obtained with an acquisition of 0.6 mm detector width, 120 kVp, reference mAs 370, 0.5 s rotation speed, 0.9 pitch and matrix size 512 × 512. The field of view varied according to the size of the harbor porpoise. Images were reconstructed to 3 mm slice thickness, 1.5 mm increment, and soft tissue algorithm (B30f medium smooth) with a window width/window level of 300/50. Images were also reconstructed to 1 mm slice thickness and an increment of 0.7 mm using a bone algorithm (B60F) with a window width/window level 3,000/600.

### 2.2 Data evaluation

All CT studies were evaluated by a veterinary diagnostic imaging resident using the viewer of the Picture Archiving and Communication System (Impax, version 6.6.1.3004, N.V., Agfa Healthcare, Mortsel, Belgium). The images were reviewed in Digital Imaging and Communication in Medicine format in the aforementioned window levels. The bony structures were evaluated using MPR. Agreement with a second observer was found in case of deviation from most common patterning (homeotic or meristic variation).

#### 2.2.1 Vertebral pattern variation

##### Vertebral column

**FIGURE 1:**
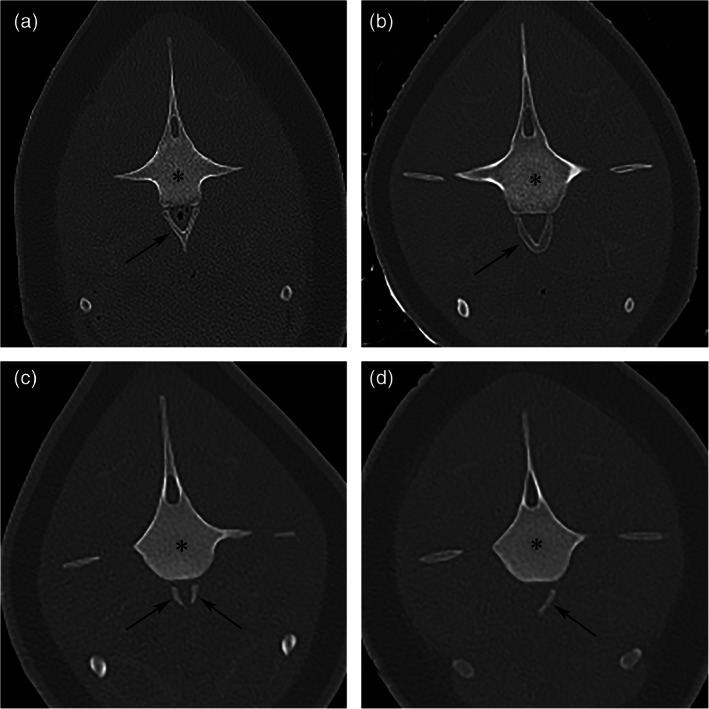
Hemal arch variation. Transverse images at the level of the most cranial hemal arch letters a–d denote the right ventral side of the images. The most cranial hemal arches (arrows) assign the first caudal vertebrae (asterisks). Bilateral pelvic bones are noted in the soft tissues ventral to the vertebrae in the soft tissues. Four different types of first hemal arches were discerned: (a) Type 1, Y‐shape. (b) Type 2, V‐shape. (c) Type 3, bilateral I‐shaped. (c) Type 4, unilateral I‐shaped

The CT images were assessed for the number of thoracic, lumbar, and caudal vertebrae per individual porpoise. Regardless of the fusion state of the syncervical, a cervical count of seven was assigned in all cases. The sum of all vertebrae combined accounted for the total vertebral count. The first thoracic vertebra was defined as the first vertebra with ribs connecting to the vertebra at the fovea costalis transversalis. The first lumbar vertebra was defined as the first vertebra without ribs. The presence of uni‐ or bilateral rib remnants in the peripheral soft tissues (floating ribs), were considered to be related to thoracic vertebrae, and thereby increasing the number of thoracic vertebrae. The presence of small, mineralized structure(s) caudoventral to the vertebral body were defined as hemal arches and defining the first caudal vertebrae (Figure 1).

##### Syncervical fusion

**FIGURE 2:**
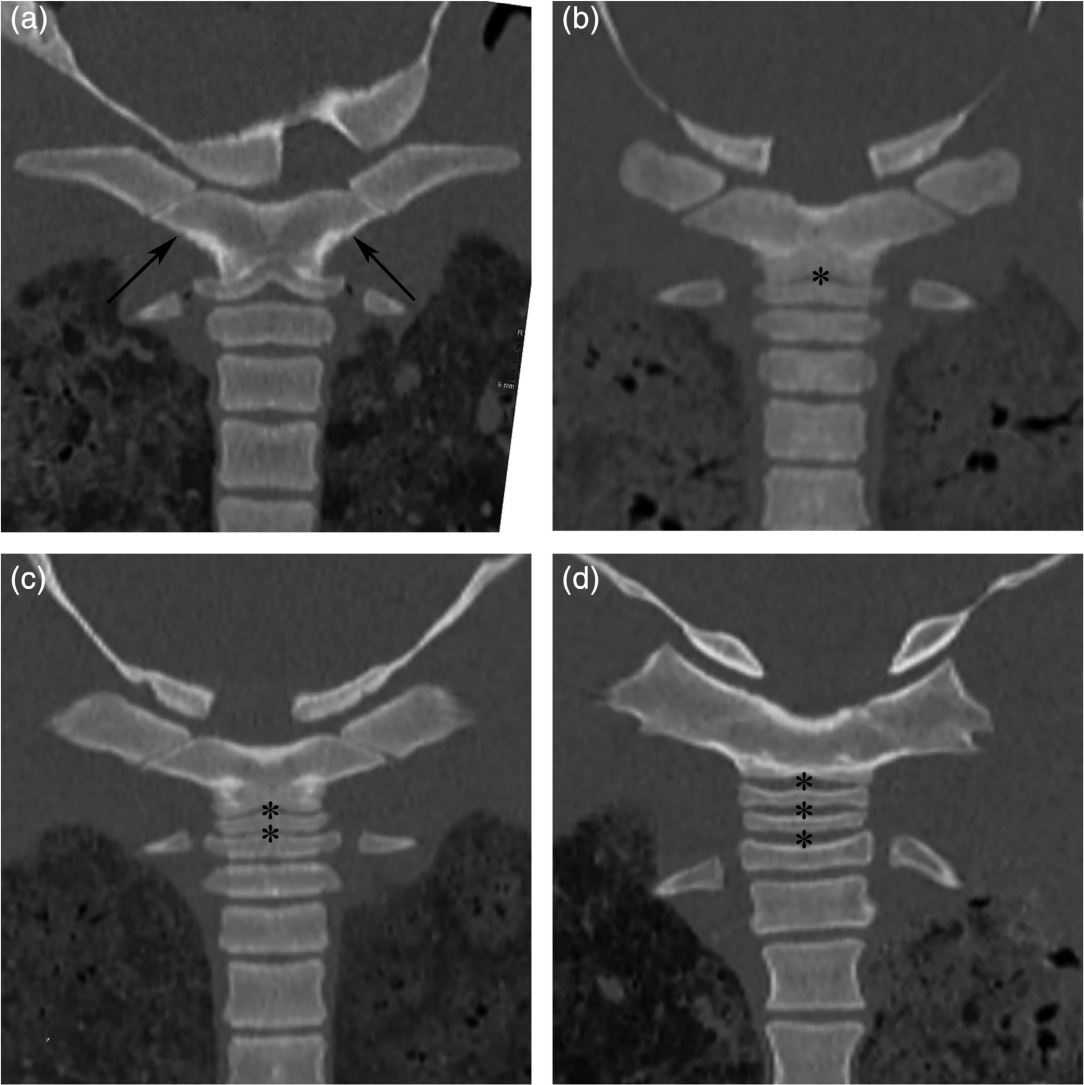
Syncervical variation. Dorsal MPR CT images, letters a–d denote the left and caudal side of the images. (a) Completely fused syncervical (arrows). Soft tissue‐attenuating zones (asterisks) separate the syncervical in, respectively, two‐part (b), three‐part (c), and four‐part (d) syncervical

Complete osseous fusion of the syncervical was assumed when there was no soft tissue‐attenuation zone, completely separating the different mineralized sections. Otherwise, the fusion state was recorded as two‐part, three‐part, or four‐part according to the number of soft tissue‐attenuating zones in between different mineralized parts of the syncervical (Figure 2).

##### Ribs

Ribs were defined as long, curved mineralized structures, of which single articulating ribs articulated at the fovea costalis transversalis of the corresponding thoracic vertebrae. Ribs were assessed for the presence of a rib head, articulating at the fovea costalis cranialis and caudalis of two adjacent vertebrae, cranial to the articulation of the rib tubercle. Those ribs were referred to as double articulating ribs. Rib remnants and ribs not articulating with transverse processes were documented as floating ribs.

##### Hemal arch

Small mineralized structures located ventral to the caudal aspect of the vertebral body were defined as hemal arches and used to define the start of the caudal vertebrae. Variation of shape of the hemal arch of the first caudal vertebra was scored as (a) Y‐shape, (b) V‐shape, (c) bilateral I‐shaped, and (d) unilateral I‐shaped (Figure 1).

### 2.3 Statistical analysis

The distribution of numerical variables was assessed for normality by the Shapiro–Wilk test and by visual inspection of quantile–quantile plots. Total body length was normally distributed and compared between adult males and females using independent sample *t*‐tests. The assumption of equal variances was tested with Levene's test. Total body length is presented as mean (standard deviation).

The distribution of number of vertebrae (per series) and fusion state of the syncervical were skewed, therefore group comparisons between sexes and age groups were made with Mann–Whitney *U*‐tests. Numbers of vertebrae (per series) are presented as median (range).

Correlations between numbers of vertebrae within each series or types of ribs were assessed by calculating the Kendall's rank correlation coefficient tau‐b (τ_b_). Kendall rank correlation is the preferred nonparametric measure of correlation for analysis of data with small sample size or ties (Arndt, Turvey, & Andreasen, 1999; Kendall, 1945).

The proportion of hemal arch type was compared between adults and immatures with Chi square test. For this analysis the symmetric and fused types (Types 1 and 2) and the incomplete/unfused types (Types 3 and 4) were combined.

For all abovementioned analyses, two‐sided tests of significance were performed with a *p*‐value <.05 considered to define statistical significance.

## 3 RESULTS

### 3.1 Vertebral column

**FIGURE 3:**
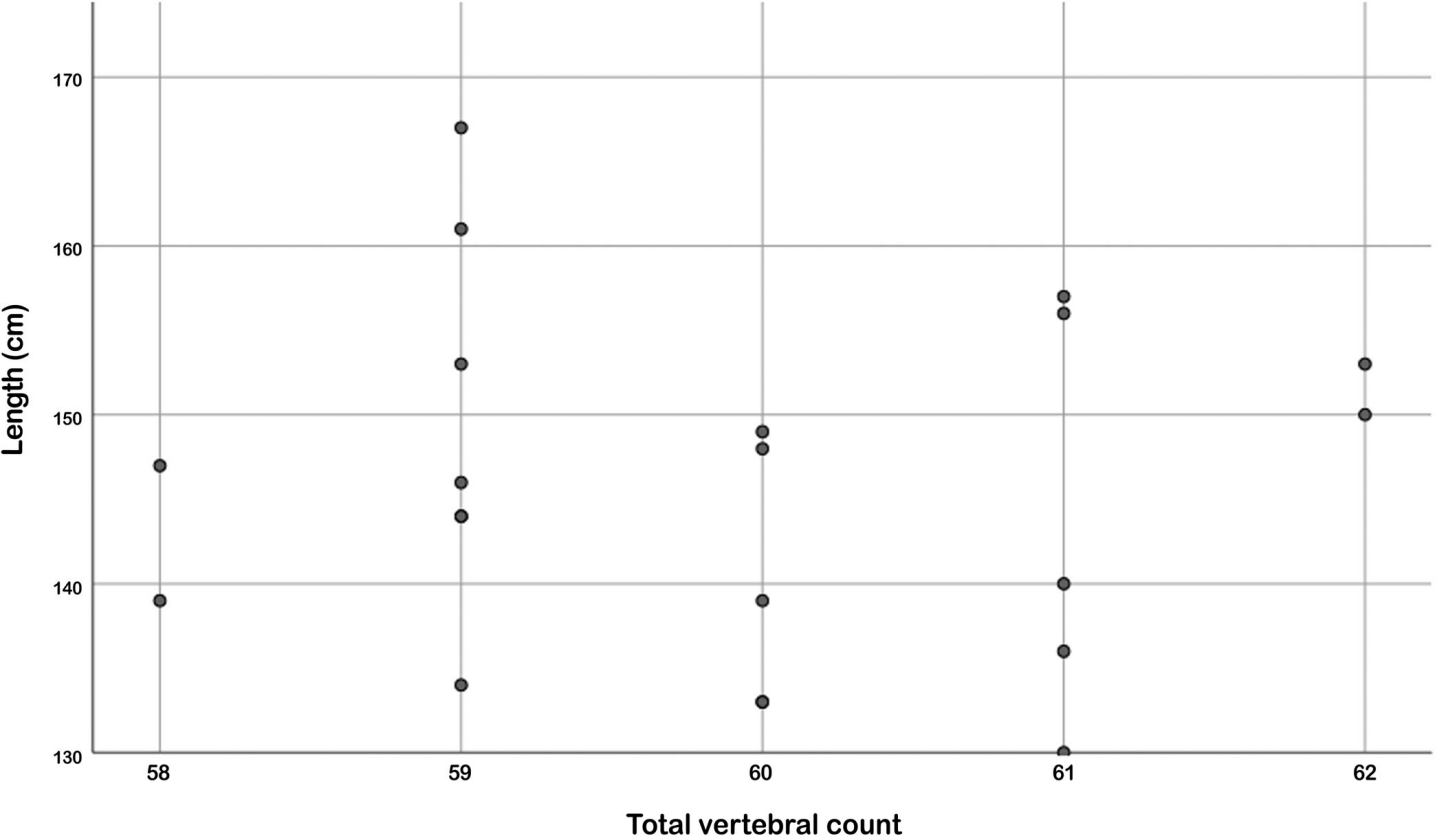
Scatter of body length variation in adults (*n* = 21) per group with similar total vertebral count. No correlation could be demonstrated using Kendall rank correlation (τ_b_ = −0.01; *p* = .98). x‐axis: total vertebral count and y‐axis: length in cm

Adult females (mean length 151.6 [SD, 10.0] cm; *n* = 10) had a significantly longer total body length compared to males (mean length 140.3 [SD, 6.5] cm; *n* = 11; *p* = .006) as measured during gross examination. The total vertebral count on CT, however, was not statistically significant different between sexes in adults (female: median, 65.5; range, 64–68; *n* = 10 and male: median, 65; range, 64–67; *n* = 11; *p* = .76). No correlation of total body length of the 21 adults with total vertebral count (τ_b_ = −0.01; *p* = .98) could be demonstrated using Kendall rank correlation (Figure 3).

**FIGURE 4:**
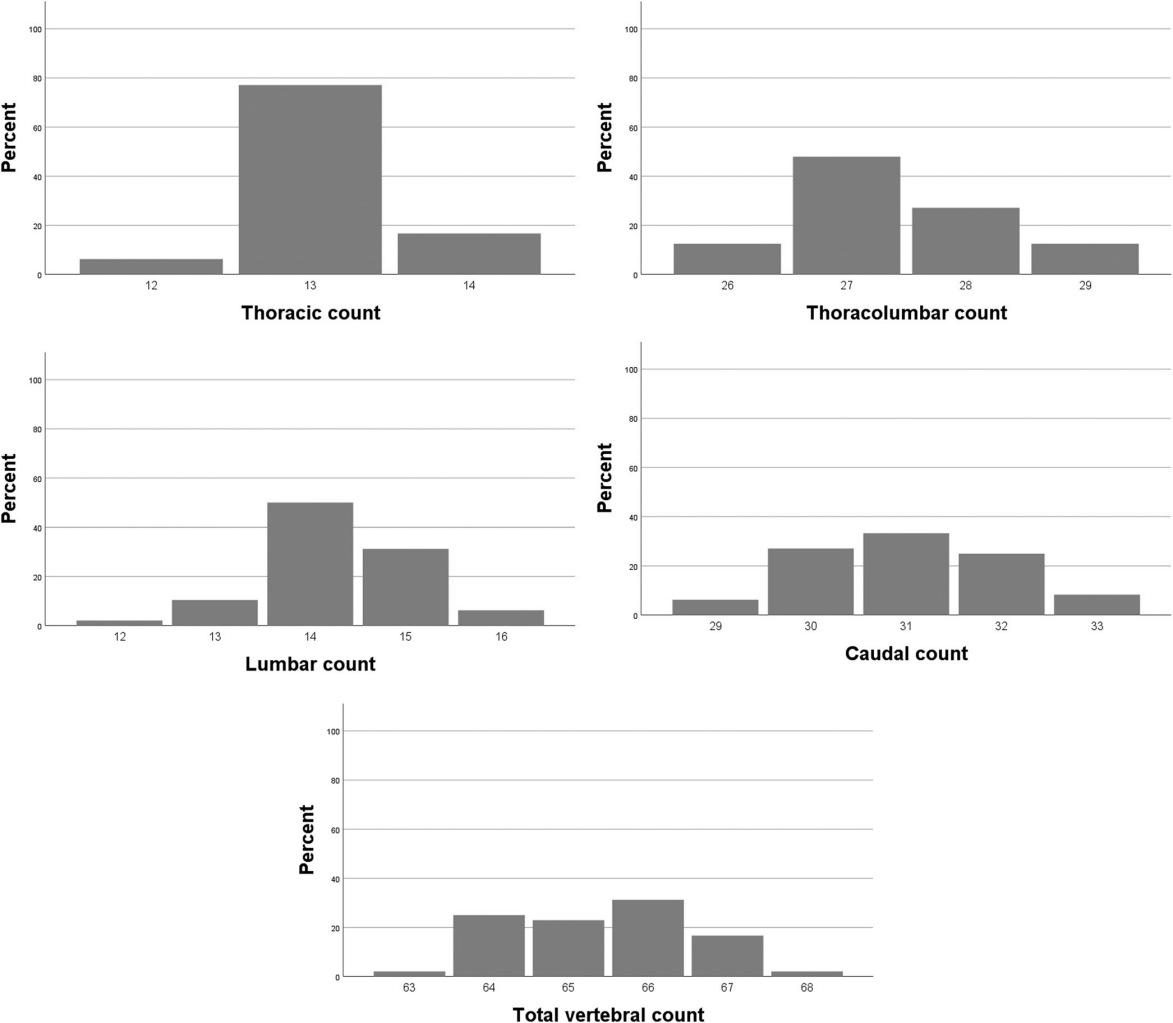
Histograms of vertebral count frequencies

The variability in vertebral formulae was high (Figure 4, Table 1). The 48 harbor porpoises showed 25 different vertebral formulas of which C7T13L14Ca30 was most common (*n* = 8/48, 17%). The thoracolumbar count ranged between 26 and 29; most common thoracolumbar count was 27 (*n* = 23/48, 48%). Using Kendall rank correlation, statistically significant positive correlations were found between total vertebral count and lumbar, thoracolumbar, and caudal vertebral count (correlation coefficients in Table 2).

**TABLE 1:** Variation in vertebral formulas, sorted by thoracolumbar count

C		T	L	Ca	Total vertebral count	No. of cases	
Thoracolumbar count = 26						6	12.5%
7		12	14	31	64	1	
7		12	14	32	65	1	
7		13	13	31	64	1	
7		13	13	32	65	1	
7		13	13	33	66	1	
7		14	12	31	64	1	
Thoracolumbar count = 27						23	48%
7		12	15	31	65	1	
7		13	14	29	63	1	
**7**		**13**	**14**	**30**	**64**	**8**	^a^
7		13	14	31	65	4	
7		13	14	32	66	4	
7		13	14	33	67	3	
7		14	13	31	65	1	
7		14	13	32	66	1	
Thoracolumbar count = 28						13	27%
7		13	15	29	64	1	
7		13	15	30	65	2	
7		13	15	31	66	5	
7		13	15	32	67	3	
7		14	14	31	66	1	
7		14	14	32	67	1	
Thoracolumbar count = 29						6	12.5%
7		13	16	30	66	1	
7		13	16	31	67	1	
7		13	16	32	68	1	
7		14	15	29	65	1	
7		14	15	30	66	2	

a Most common vertebral formula C7T13L14Ca30.

**TABLE 2:** Association with total vertebral count, using Kendall rank correlation

	Association with total vertebral count	
	Correlation coefficient tau‐b (τ_b_)	*p*‐Value
Thoracolumbar count	.42	.001
Thoracic count	.14	.30
Lumbar count	.35	.006
Caudal count	.61	<.001

*Note*: Thoracolumbar, lumbar, and caudal count are positively associated with total vertebral count (*p*‐value <.05).

### 3.2 Syncervical fusion

**FIGURE 5:**
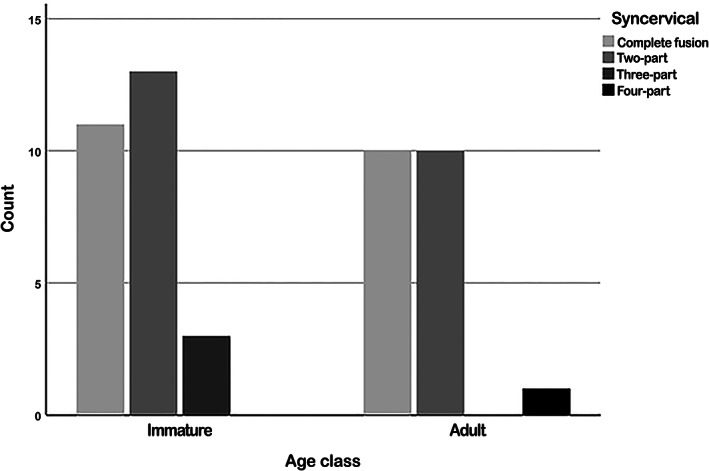
Histograms of syncervical variation

The syncervical was classified as completely fused in 44% (*n* = 21/48), two‐part in 48% (*n* = 23/48), three‐part in 6% (*n* = 3/48), and four‐part in 2% (1/48) (Figures 2 and 5). The three‐part syncervical was only found in immature harbor porpoises. A four‐part syncervical was seen in one adult harbor porpoise. No statistical difference in fusion state was identified between adult and immature harbor porpoises (*p* = .54), using Mann–Whitney *U*‐tests.

### 3.3 Ribs

The medial part of the ribs and the transverse processes in immatures showed reduced visibility, due to incomplete mineralization creating poor contrast at the level of rib articulation. Therefore, immature harbor porpoises were excluded from the rib analyses.

There were 10 adult harbor porpoises without floating ribs, with thoracic count 13 and lumbar count range 14–16. There were nine harbor porpoises with one vertebra with floating ribs (of which one harbor porpoise had a unilateral floating rib, and no rib visible on the contralateral side), with thoracic count range 13–14 and lumbar count range 13–15. And there were two harbor porpoises with two vertebrae with floating ribs (of which one harbor porpoise had bilateral floating ribs at the level of T13, a unilateral floating rib at the level of T14, and no rib visible on the contralateral side), with thoracic count 14 and lumbar count range 12–15. The number of vertebrae with floating ribs in adults was negatively associated with number of thoracic vertebrae with a connected rib (τ_b_ = −0.80; *p* < .001), but not with lumbar vertebral count (τ_b_ = −0.10; *p* = .64).

In most adults, the cranial seven thoracic vertebrae had double articulating ribs (of which two harbor porpoises had a unilateral double articulating seventh rib, and the contralateral ribs were single articulating at the fovea costalis transversalis). Two adult harbor porpoises had six double articulating ribs and five adult harbor porpoises had eight double articulating ribs (of which one harbor porpoise had a unilateral double articulating eighth rib, and the contralateral rib was single articulating at the fovea costalis transversalis). The remainder articulated only at the fovea costalis transversalis of the transverse processes or was defined as floating ribs. The number of double articulating ribs was not correlated with thoracic count (τ_b_ = 0.14; *p* = .52).

### 3.4 Hemal arches

**FIGURE 6:**
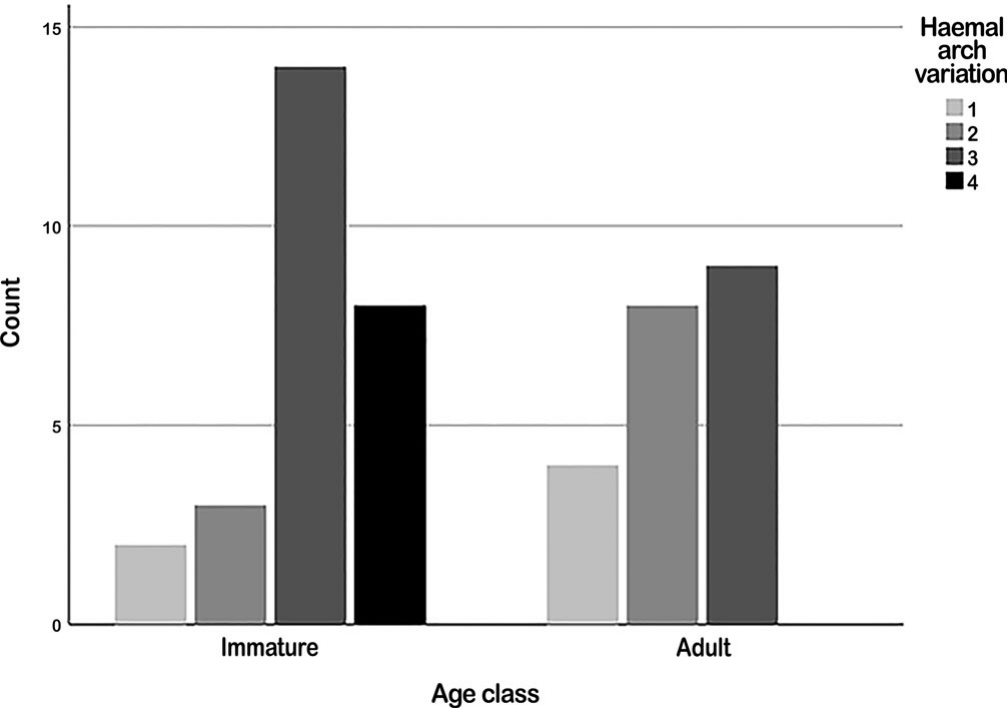
Histograms of hemal arch variation

The presentation of the first hemal arch varied greatly (Figures 1 and 6).

Type 1 hemal arch was present in 13% of harbor porpoises (immatures: *n* = 2/27, 7%; adult: *n* = 4/21, 19%), type 2 hemal arch in 23% (immatures: *n* = 3/27, 11%; adult: *n* = 8/21, 38%), type 3 in 48% (immatures: *n* = 14/27, 52%; adult: *n* = 9/21, 43%) and type 4 in 17% (immatures: *n* = 8/27, 30%; adult: *n* = 0/21, 0%).

A statistically significant difference was found with hemal arch type 3 and 4 being more common in immature harbor porpoises, and hemal arch types 1 and 2 more common in adult harbor porpoises (*p* = .007).

## 4 DISCUSSION

This study showed a high degree of homeotic and meristic variation in the thoracic, lumbar, and caudal vertebral series. There were 25 different vertebral patterns identified, of which formula C7T13L14Ca30 (*n* = 8, 17%) was most common. This included the previous reported vertebral formulas C7T13L14Ca31 (Buchholtz et al., 2005) and C7T12L15Ca30–35 (Galatius & Kinze, 2003). Total vertebral count of the porpoises in this study varied from 63 to 68, showing meristic variation. As shown in Figure 4, the degree of variation in vertebral count per series increased in a craniocaudal direction, with most variation in number of vertebrae identified in the caudal vertebral series. The variation in total vertebral count was associated with variation in number of caudal and lumbar vertebrae (Table 2). Thoracolumbar count was varying between 26 and 29 vertebrae (Table 1). Therefore, vertebral count in the harbor porpoise is not as conservative as seen in terrestrial mammals (Galis, 1999; Galis et al., 2006; Narita & Kuratani, 2005).

In addition to variation in total vertebral count, variation in the number of vertebrae in two adjacent series in harbor porpoises with identical total vertebral count was found (Table 1), suggesting homeotic variation in the thoracic, lumbar and caudal vertebral series. For example, a harbor porpoise with vertebral formula C7T12L15Ca31 possesses a transitional vertebra with lumbarization of T13, when compared to the most common vertebral formula of C7T13L14Ca30. And a harbor porpoise with vertebral formula C7T13L15Ca29 possesses a transitional vertebra with lumbarization of Ca1, when compared to the most common vertebral formula of C7T13L14Ca30.

Four different types of first hemal arches were distinguished (Figure 1). Types 1 and 2 are similar to two out of five cranial view morphotypes in mammalian species, as described by Zavodszky and Russo (2020). As types 1 and 2 are symmetric and fused in the center, they were considered more mature and complete variations. This is supported by the finding that adult harbor porpoises had significantly more often hemal arch types 1 and 2 and immatures had significantly more often hemal arch types 3 and 4. The high presence of type 3 hemal arch in adult harbor porpoises (9/21, 43%) might be explained by the occurrence of pedomorphosis, in which immature characteristics are seen in adult animals. Evidence of pedomorphosis has been observed in other skeletal elements of harbor porpoises, such as the vertebral epiphysis (Galatius & Kinze, 2003) and digits (Galatius, Andersen, Haugan, Langhoff, & Jespersen, 2006).

In many cetaceans, two or more of the seven cervical vertebrae are fused (Buchholtz et al., 2005; Buchholtz & Schur, 2004; Cozzi et al., 2017; Newcomer et al., 1996; Nishiwaki, 1963; VanBuren & Evans, 2017). Buchholtz and Schur (2004) describe variation of the number of vertebrae included in the fusion of the syncervical among individuals of the same species of Delphinidae. This is the first study, showing variation in syncervical fusion in the harbor porpoise. A single, completely fused syncervical (43%, *n* = 21/48) and a two‐part syncervical (48%, *n* = 23/48) are common in this group of harbor porpoise. Three‐part (6%, *n* = 3/48) and four‐part (2%, *n* = 1/48) syncervical variations also occurred. Nishiwaki (1963) described different genera of Delphinidae having fused atlas and axis, and considered the third cervical vertebra to fuse with the axis after growth to maturity in the *Stenella* and *Delphinus* (Nishiwaki, 1963). Progressive fusion of the syncervical during life, representing ontogenetic variation could explain the presence of different fusion states. However, no statistically significant correlation of fusion status with age class was found in the current study. Moreover, the only four‐part syncervical was found in an adult female harbor porpoise. In addition to hemal arch type 3, the incompletely fused syncervical in adults could be considered as pedomorphic characteristics.

Adult females (mean, 151.6 [SD, 10.0] cm; *n* = 10) had a longer total body length compared to males (mean, 140.3 [SD, 6.5] cm; *n* = 11; *p* = .006), in accordance to the biological difference within this species where females tend to grow longer than males (Galatius, 2005; Lockyer, Heide‐Jørgensen, Jensen, Kinze, & Buus Sørensen, 2001). Because no association of sex with difference in vertebral count could be found, the greater length of females is most likely due to larger vertebrae than more vertebrae.

First, a limitation of this study is the difficulty in implementation of documented skeletal variation, for there is no absolute knowledge considering standard vertebral formula and morphology of hemal arches and syncervical. Secondarily, in the absence of data on absolute age, the differentiation between immature and adult stranded harbor porpoises at the Division of Pathology is routinely made by assessment of the reproductive organs (IJsseldijk et al., 2019). In addition, we assessed closure of the proximal humeral physes. CT examination of the proximal humeral physis might be a reliable and fast way to confirm the age class of a harbor porpoise carcass. To confirm CT examination of the humeral physes as tool for age estimation, future studies could be focused on comparison with tooth structure analysis (Evans, Kemper, McKenzie, & McIntosh, 2001), for this is considered a more reliable method than age estimation by reproduction status. In the third place, the high degree of variation in the vertebral formula and the small sample size limited statistical power.

CT examination of stranded harbor porpoises shows high vertebral formula variation within species, including homeotic and meristic variation. Additionally, variation was found in fusion state of the syncervical and morphology of the first hemal arch, which might represent pedomorphic features.

## AUTHOR CONTRIBUTIONS

**Dorien S. Willems:** Conceptualization; data curation; formal analysis; investigation; methodology; project administration; supervision; validation; visualization; writing‐original draft. **Lonneke L. IJsseldijk:** Conceptualization; data curation; investigation; writing‐original draft. **D. Hendrik N. van den Broek:** Formal analysis; methodology; validation; writing‐original draft. **Stefanie Veraa:** Conceptualization; formal analysis; investigation; methodology; supervision; writing‐original draft.

## Supporting information


**Figure S1** Supporting Information.

**Table S1** Supporting Information.
