# Supplementary figures and images for: Vertebral pattern variation in the North Sea harbor porpoise (Phocoena phocoena) by computed tomography
Source: Anat Rec (Hoboken). 2020 Oct 16;304(5):968–78. doi: 10.1002/ar.24524 (PMC8246778; doi:10.1002/ar.24524)

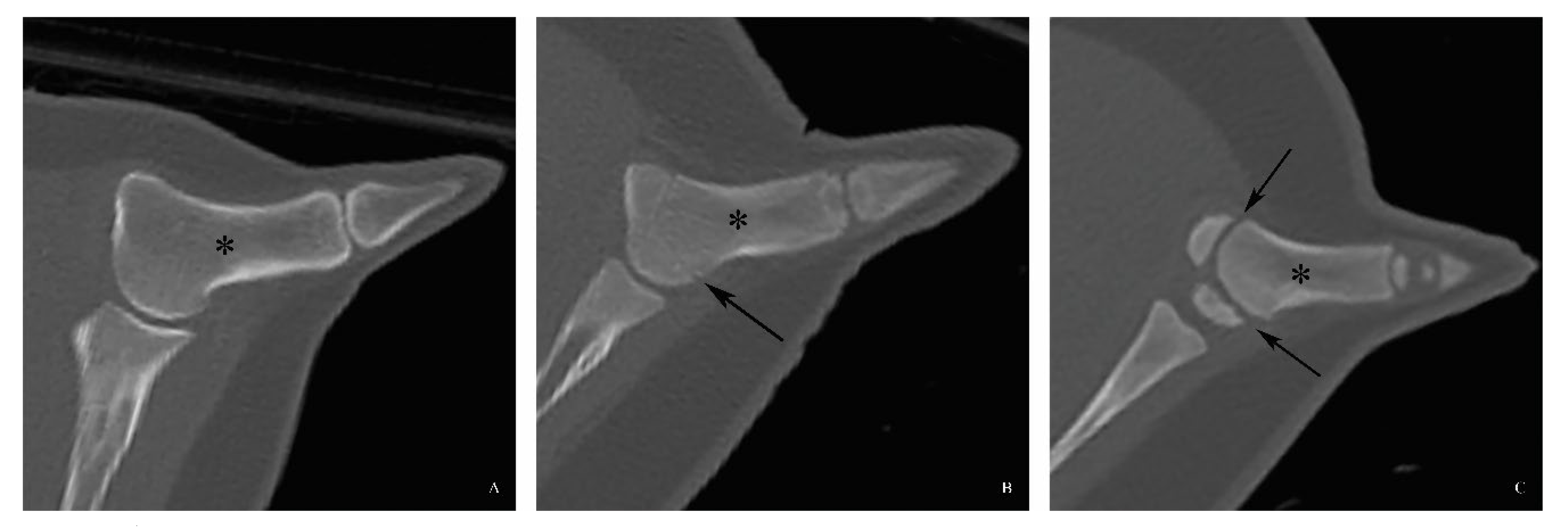

Supplement: Supplementary file 1 — Figure S1 Supporting Information. [file AR-304-968-s002.tif]
