# Supplementary material for: Vertebral pattern variation in the North Sea harbor porpoise (Phocoena phocoena) by computed tomography
Source: Anat Rec (Hoboken). 2020 Oct 16;304(5):968–78. doi: 10.1002/ar.24524 (PMC8246778; doi:10.1002/ar.24524)
Supplement: Supplementary file 2 — Table S1 Supporting Information. [file AR-304-968-s001.docx]

|  | **Sex** | **Length (cm)** | **Age class** | **Physes proximal humerus** | **Cervical fusion state** | **Haemal arch variation** | **Thoracic count with double articulating rib(s)** | **Thoracic count with connected rib(s)** | **Thoracic count with floating rib (s)** | **Cervical count** | **Thoracic count** | **L** | **Cd** | **Total count** |
| --- | --- | --- | --- | --- | --- | --- | --- | --- | --- | --- | --- | --- | --- | --- |
| #1 | male | 130 | Adult | closed | complete | 3 | 8 | 12 | 1 | 7 | 13 | 15 | 32 | 67 |
| #2 | male | 144 | Adult | closed | two-part | 2 | 7 | 12 | 1 | 7 | 13 | 14 | 30 | 64 |
| #3 | male | 110 | Immature | open | complete | 3 |  | 13 |  | 7 | 14 | 14 | 31 | 66 |
| #4 | male | 110 | Immature | open | complete | 3 |  | 12 |  | 7 | 12 | 15 | 31 | 65 |
| #5 | male | 133 | Adult | closed | complete | 2 | 8 | 12 | 1 | 7 | 13 | 15 | 31 | 66 |
| #6 | female | 100 | Immature | open | two-part | 3 |  | 12 |  | 7 | 13 | 15 | 31 | 66 |
| #7 | male | 128 | Immature | closed | two-part | 2 |  | 12 |  | 7 | 13 | 14 | 29 | 63 |
| #8 | male | 150 | Adult | closed | two-part | 2 | 8 | 13 | 0 | 7 | 13 | 15 | 32 | 67 |
| #9 | male | 115 | Immature | open | complete | 3 |  | 11 |  | 7 | 12 | 14 | 31 | 64 |
| #10 | female | 153 | Adult | closed | complete | 2 | 8 | 13 | 0 | 7 | 13 | 16 | 32 | 68 |
| #11 | male | 134 | Adult | closed | two-part | 2 | 7 | 13 | 0 | 7 | 13 | 14 | 30 | 64 |
| #12 | male | 115 | Immature | open | complete | 3 |  | 12 |  | 7 | 13 | 14 | 32 | 66 |
| #13 | female | 134 | Immature | open | complete | 4 |  | 12 |  | 7 | 14 | 13 | 31 | 65 |
| #14 | female | 133 | Adult | closed | two-part | 3 | 7 | 13 | 0 | 7 | 13 | 14 | 31 | 65 |
| #15 | female | 139 | Adult | closed | complete | 1 | 7 | 13 | 1 | 7 | 14 | 15 | 30 | 66 |
| #16 | female | 149 | Adult | closed | complete | 2 | 6 | 13 | 0 | 7 | 13 | 14 | 32 | 66 |
| #17 | male | 144 | Adult | closed | complete | 2 | 7 | 12 | 1 | 7 | 13 | 14 | 31 | 65 |
| #18 | male | 79 | Immature | open | complete | 3 |  | 13 |  | 7 | 13 | 16 | 30 | 66 |
| #19 | male | 80 | Immature | open | two-part | 4 |  | 13 |  | 7 | 13 | 14 | 33 | 67 |
| #20 | male | 117 | Immature | open | two-part | 4 |  | 12 |  | 7 | 13 | 14 | 31 | 65 |
| #21 | male | 147 | Adult | closed | complete | 3 | 7 | 12 | 2 | 7 | 14 | 12 | 31 | 64 |
| #22 | female | 148 | Adult | closed | complete | 2 | 7 | 13 | 1 | 7 | 14 | 13 | 32 | 66 |
| #23 | female | 113 | Immature | open | complete | 2 |  | 11 |  | 7 | 13 | 14 | 30 | 64 |
| #24 | female | 167 | Adult | closed | two-part | 3 | 7 | 12 | 1 | 7 | 13 | 13 | 31 | 64 |
| #25 | male | 98 | Immature | open | two-part | 3 |  | 12 |  | 7 | 13 | 15 | 30 | 65 |
| #26 | male | 113 | Immature | open | two-part | 3 |  | 12 |  | 7 | 13 | 14 | 33 | 67 |
| #27 | female | 161 | Adult | closed | two-part | 3 | 7 | 13 | 0 | 7 | 13 | 14 | 30 | 64 |
| #28 | male | 136 | Adult | closed | two-part | 1 | 7 | 12 | 1 | 7 | 13 | 15 | 31 | 66 |
| #29 | female | 156 | Adult | closed | two-part | 1 | 8 | 13 | 0 | 7 | 13 | 15 | 31 | 66 |
| #30 | female | 84 | Immature | open | complete | 3 |  | 12 |  | 7 | 13 | 14 | 32 | 66 |
| #31 | female | 153 | Adult | closed | complete | 3 | 7 | 13 | 0 | 7 | 13 | 14 | 31 | 65 |
| #32 | female | 111 | Immature | open | two-part | 3 |  | 12 |  | 7 | 13 | 15 | 31 | 66 |
| #33 | male | 139 | Adult | closed | complete | 3 | 7 | 13 | 0 | 7 | 13 | 14 | 30 | 64 |
| #34 | female | 157 | Adult | closed | four-part | 3 | 7 | 12 | 1 | 7 | 13 | 14 | 30 | 64 |
| #35 | male | 146 | Adult | closed | two-part | 1 | 6 | 13 | 0 | 7 | 13 | 14 | 30 | 64 |
| #36 | male | 112 | Immature | open | three-part | 4 |  | 12 |  | 7 | 14 | 14 | 32 | 67 |
| #37 | male | 115 | Immature | open | complete | 4 |  | 13 |  | 7 | 13 | 15 | 32 | 67 |
| #38 | male | 140 | Adult | closed | two-part | 3 | 7 | 12 | 2 | 7 | 14 | 15 | 30 | 66 |
| #39 | male | 113 | Immature | open | three-part | 3 |  | 12 |  | 7 | 13 | 13 | 32 | 65 |
| #40 | male | 113 | Immature | open | complete | 4 |  | 12 |  | 7 | 13 | 14 | 33 | 67 |
| #41 | male | 100 | Immature | open | two-part | 3 |  | 12 |  | 7 | 12 | 14 | 32 | 65 |
| #42 | female | 109 | Immature | open | two-part | 1 |  | 12 |  | 7 | 14 | 15 | 29 | 65 |
| #43 | male | 112 | Immature | open | two-part | 4 |  | 12 |  | 7 | 13 | 13 | 33 | 66 |
| #44 | female | 110 | Immature | open | two-part | 4 |  | 13 |  | 7 | 13 | 14 | 32 | 66 |
| #45 | female | 111 | Immature | open | two-part | 1 |  | 13 |  | 7 | 13 | 16 | 31 | 67 |
| #46 | female | 102 | Immature | open | three-part | 2 |  | 12 |  | 7 | 13 | 15 | 30 | 65 |
| #47 | female | 72 | Immature | open | complete | 3 |  | 13 |  | 7 | 13 | 15 | 29 | 64 |
| #48 | female | 91 | Immature | open | two-part | 3 |  | 13 |  | 7 | 13 | 14 | 30 | 64 |
